# Effects of Medetomidine, Dexmedetomidine and their combination with Acepromazine on the intraocular pressure (IOP), tear secretion and pupil diameter in dogs

**DOI:** 10.1002/vms3.467

**Published:** 2021-03-10

**Authors:** Ali Aghababaei, Ali Ronagh, Bahman Mosallanejad, Ali Baniadam

**Affiliations:** ^1^ Graduate of Doctor of Veterinary Medicine Faculty of Veterinary Medicine Shahid Chamran University of Ahvaz Ahvaz Iran; ^2^ Department of Clinical Sciences Faculty of Veterinary Medicine Shahid Chamran University of Ahvaz Ahvaz Iran

**Keywords:** Acepromazine, Dexmedetomidine, dog, intraocular pressure (IOP), Medetomidine

## Abstract

**Background:**

A great number of sedatives and anaesthetics have been used to perform surgeries or routine ophthalmologic examinations in animals and sometimes the combination of these medicines has more suitable effects than each one alone.

**Objectives:**

This paper aims to explore the main effects of Medetomidine + Acepromazine, Dexmedetomidine + Acepromazine on intraocular pressure, tear secretion and pupil diameter.

**Methods:**

To accomplish the aforementioned aim, 32 adult dogs (aged one‐to‐three‐years‐old) were clinically examined. Dogs were divided into four groups consisting of group DA, Dexmedetomidine (5 µg/kg) + Acepromazine (0.05 mg/kg); Group D, Dexmedetomidine (5 µg/kg); Group M, Medetomidine (10 µg/kg); Group MA, Medetomidine (10 µg/kg) + Acepromazine (0.05 mg/kg). The ocular factors including tear production, pupil diameter and intraocular pressure of both right and left eyes were first measured and then recorded in each dog at time T_0_ (−15 min). Afterwards, the drugs were administered intramuscularly, based on which the ocular factors were re‐measured at T_1_ (+5 min), T_2_ (+15 min) and T_3_ (+20 min). All four groups showed a reduction in intraocular pressure, which was significant in DA, D and M groups.

**Results:**

Furthermore, there was a fluctuation in the amount of tear secretion in DA and D groups (increase and then decrease), as well as a significant reduction in M and MA groups. Decreasing in pupil diameter also occurred in all four groups, but the reduction was significant only in DA and MA groups.

**Conclusion:**

According to the results obtained, as the changes caused by the systemic administration of the above drug compounds did not exceed the physiological range, it can be concluded that these combinations could be utilized as suitable sedatives or pre‐anaesthetic compounds in the eye surgeries.

## Graphical abstract

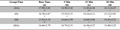

## 1 INTRODUCTION

Eye examinations in animals, including dogs, are difficult because of their resistance. Therefore, sedatives have the potential to reduce the animals' stress during the examination and facilitate the examination, so that there will be less risk for both patient and examiner (Dodam et al., 1998). Furthermore, the use of some sedatives can reduce doses of injectable and inhalational anaesthetics and they also have antiemetic and anti‐arrhythmic properties (Monteiro et al., 2016). A number of sedatives, including alpha‐2 agonists, narcotics, phenothiazines and benzodiazepines, have been used alone or in combination to create sedative effects for surgery or routine examinations (Interlandi et al., 2017; Rankin, 2015). Medetomidine is an alpha‐2 adrenoceptor that is used to create relaxation and pain relief. It is more specific to receptors than xylazine and has a greater pharmacological tendency to alpha‐2 receptors (Sawyer, 2008). Dexmedetomidine is a 2A‐adrenoceptor and is actually an isomer of Medetomidine, which is very similar to Medetomidine in terms of pharmacokinetic properties in dogs (Rankin, 2015). Acpromazine is a mild to moderate phenothiazine sedative in dogs. In addition to its sedative properties, by interfering with dopamine transfer in the central nervous system (CNS), it can reduce the dose of anaesthetic injections and inhalers, and has anti‐emetic and anti‐arrhythmic properties (Monteiro et al., 2009). Studies have indicated that some of these drugs and combinations have ocular side effects (Leonardi et al., 2020; Schroder et al., 2018). This study aimed to investigate the combined effects of Medetomidine and Acepromazine as well as Dexmedetomidine and Acepromazine on intraocular pressure, tear secretion and pupil diameter.

## 2 MATERIALS AND METHODS

First, It should also be noted that the project was approved by the local Committee of the Institutional Animal Care and Use of *** (no. ***).

Thirty‐two adult dogs aged between one and 3 years‐old were selected from mixed breeds. These dogs were kept at the university shelter. Before the study, the dogs were examined in terms of non‐pregnancy. In addition, physical examination and ophthalmic examination including indirect ophthalmoscopy, fluorescein staining and STT were performed. The animals were classified into four 8‐dog groups, including the following groups :Group D: Dexmedetomidine (5 µg/kg), Group M: Medetomidine (10 µg/kg), Group DA: The combination of Dexmedetomidine (5 µg/kg) and Acepromazine (0.05 mg/kg), Group MA: The combination of Medetomidine (10 µg/kg) and Acepromazine (0.05 mg/kg) (Monteiro et al., 2008, 2009; Raszplewicz et al., 2013).

First, each dog was placed in the study room for 2 hr to adapt to environmental conditions, including light, and then the eye factors were measured and recorded in both the right and left eyes of each dog at time T_0_ (−15 min). The necessary amount of drug was calculated according to the weight of animals and its volume was diluted with normal saline to 2 ml. The drugs were then intramuscularly injected into semitendinosus muscle and the ocular factors were re‐measured at time T_1_ (+5 min), T_2_ (+15 min) and T_3_ (+20 min) (Santos et al., 2013). Today, despite the invention of advanced instruments for measuring intraocular pressure, the Schiotz tonometer is still used. So in this study, Schiotz tonometer was used to measure intraocular pressure (Estrovich et al., 2015). The calliper was used to measure the pupil diameter. For this purpose, the calliper was adjusted according to the pupil diameter oriented parallel to the corneal surface of the eye, but without touching the cornea. The amount of tear secretion was also assessed using Schirmer's paper tape. The animal was kept in sternal recumbency. The end of the sterile Schirmer strip was extracted from the package and folded, the calibrated part is still in the package, and placed into the lower conjunctival fornix of the eye for one min. During this time, the eyelids remained closed. Food and water were withheld, respectively, 12 and 2 hr before the procedure (Santos et al., 2013).

Data of the right and left eyes were calculated and considered for each animal on average and were evaluated in SPSS. One‐way analysis of variance was used to analyse and compare the data between the studied groups. Also, the analysis of variance test (ANOVA) with repeatability and LSD posttest were used to compare intragroup data. The results were presented as the mean of the standard deviation and *p* < .05 was considered statistically significant.

## 3 RESULTS

The left and right eyes of all dogs were thoroughly examined prior to the study, and dogs with two healthy eyes were tested. On the other hand, this study aimed to investigate the overall effect of the drug on ocular factors, not to compare the right and left eyes; hence, data on the right and left eyes were calculated and considered for each animal on average.

The baseline values of intraocular pressure were 17.99 ± 3.02, 16.78 ± 3.67, 17.63 ± 3.19 and 16.66 ± 2.79, respectively, in dogs in all four groups, DA, D, M and MA that were in the normal range and did not differ significantly from each other. There was a decrease in intraocular pressure after the intramuscular administration of the drug during the first 5 min in Group DA, and the pressure reduction continued until 20 min after the injection. The reduction was significant at 15 and 20 min compared to the baseline time (*p* < .05). Reduction in intraocular pressure was also seen in Group D at 5–20 min and the results indicated a significant difference between baseline times and 20 min after Dexmedetomidine injection (*p* < .05). In the third group of the study (Group M), the injection of Medetomidine, like the previous two groups, caused a decrease in intraocular pressure and showed a significant difference between 15 and 20 min after injection, compared to the baseline pressure (*p* < .05), but after intramuscular injection of Medetomidine‐Acepromazine in the fourth group (Group MA), a slight increase in intraocular pressure was observed at +5 min, and then a decrease was seen in the intraocular pressure at 15 and 20 min., which was lower than the baseline time, but the difference was not significant (as depicted in Table 1).

**TABLE 1:** Mean values ± standard error of intraocular pressure (IOP, mm Hg)

Group\time	Base time (a)	5 min (b)	15 min (c)	20 min (d)
(DA)	17.99 ± 3.02^cd^	14.50 ± 5.42	11.03 ± 6.36^a,D^	10.11 ± 6.87^a^
(D)	16.78 ± 3.67^d^	15.53 ± 5.31^d^	14.16 ± 4.11	13.12 ± 5.83^ab^
(M)	17.63 ± 3.19^cd^	16.66 ± 2.99	14.68 ± 5.04^a^	14.43 ± 4.52^a^
(MA)	16.66 ± 2.79	16.75 ± 2.31	16.58 ± 4.27^A^	15.40 ± 3.25

Note

The lowercase letter indicates a significant difference in each group (*p* < .05). The capital letter indicates a significant difference between the groups (*p* < .05).

According to the comparison of results obtained from the groups, there was a significant difference between DA and MA groups in terms of intraocular pressure only +15 min after the drug injection (*p* < .05). The lowest intraocular pressure of 10.11 ± 6.87 was seen in the Group DA at +20 min. The amounts of tear secretion for the groups were 14.64 ± 2.56, 15.25 ± 1.66, 14.43 ± 1.11 and 14.56 ± 2.88, respectively, in the Schirmer's test at the baseline time. The values were approximately equal to the minimum base level (15 mm/min) mentioned in the references (Maggs et al., 2008). On the other hand, the dogs did not show any signs of discoloration in the cornea or Keratoconjunctivitis sicca. In Group DA, the tear secretion process decreased until +15 min, and then increased in the 20th min onwards after Dexmedetomidine‐Acepromazine injection compared to +15 min, but it was still lower than the baseline. In these decreasing and then increasing trends, there was no significant difference between results of various times of group. In the second group, which used Dexmedetomidine, the tear secretion amount first had a reduction and then an increase. At +5 min, compared to the baseline, the amount of tear secretion decreased, but it started to increase from the 15th min, so that it exceeded the baseline at +20 min, and even showed a significant difference compared to the amount of tear secretion at +5 min (*p* < .05). In Group M, the amount of tear secretion clearly and significantly decreased, so that it showed a significant difference from the baseline at +5, +15 and +20 min (*p* < .05). In Group MA, injection of a combination of Medetomidine + Acepromazine significantly decreased the eye tear secretion at all three times (*p* < .05). There was also a significant difference between values obtained at 5 and 20 min after injection in the group (*p* < .05).

Comparison of groups at the baseline did not show any difference between the values, but the result obtained in the group MA showed a significant difference in the decreasing trend compared to other groups at the 5th min after the injection (*p* < .05). At +15 min, there was a significant difference between groups DA and D with group MA like +5 min (*p* < .05). The results also indicated a significant difference between values of group D with group M at 15 min after the drug injection (*p* < .05). At +20 min, there was no significant difference in values between the groups DA and D, but separately, individual differences were noted with groups M and MA (*p* < .05) (as shown in Table 2).

**TABLE 2:** Mean values ± standard error of tear secretion (Schirmer 1 Tear Test) (mm/min)

Group\time	Base time (a)	5 min (b)	15 min (c)	20 min (d)
(DA)	14.64 ± 2.56	13.25 ± 1.80^D^	12.87 ± 1.70^D^	13.12 ± 2.46^CD^
(D)	15.25 ± 1.66	13.57 ± 1.96^d,D^	14.35 ± 2.42^CD^	15.64 ± 1.62^b,CD^
(M)	14.43 ± 1.11^bcd^	12.33 ± 3.41^a,D^	8.78 ± 3.79^a,B^	7.00 ± 2.03^a,AB^
(MA)	14.56 ± 2.88^bcd^	8.66 ± 1.53^ad,ABC^	8.25 ± 3.52^a,AB^	5.92 ± 2.50^ab,AB^

Note

The lowercase letter indicates a significant difference in each group (*p* < .05). The capital letter indicates a significant difference between the groups (*p* < .05).

Values related to the size of pupil diameter in the group DA followed a downward trend, so that a significant difference with the baseline was seen at +20 min (*p* < .05). The reduction was also seen in the group MA in a way that the baseline and +5 min did not show any significant change, but +15 and +20 min had a significant decrease compared to the baseline and +5 min (*p* < .05). In groups D and M, there was no significant change in size of pupil diameter and no significant difference was observed between the baseline time and other times in the animals. The results of groups in this study indicated that the lowest pupil diameter belonged to the group MA at +20 min. The value showed a significant difference from group MA (*p* < .05), but no significant difference was seen between the groups in other cases (as illustrated in Table 3).

**TABLE 3:** Mean values ± standard error of pupil diameter (mm)

Group\time	Base time (a)	5 min (b)	15 min (c)	20 min (d)
(DA)	11.03 ± 1.67^d^	10.45 ± 2.45	10.04 ± 2.72	9.50 ± 2.75^a^
(D)	11.98 ± 1.20	11.58 ± 1.22	11.38 ± 0.91	11.30 ± 1.04^D^
(M)	10.87 ± 1.03	11.00 ± 1.45	10.82 ± 1.25	10.81 ± 0.92
(MA)	11.23 ± 1.42^cd^	11.15 ± 1.24^cd^	10.03 ± 1.98^ab^	9.06 ± 2.64^ab,B^

Note

The lowercase letter indicates a significant difference in each group (*p* < .05). The capital letter indicates a significant difference between the groups (*p* < .05).

## 4 DISCUSSION

In this study, there was a reduction in the intraoccular pressure in the MA group. However, the parameter had a significant decrease at the 15th and 20th min in the M group, compared to baseline time, but it was not significant in the MA group. In a study by Schroder et al. (2018) that investigated the effects of tramadol and Acepromazine and their combination on dog eye factors, they found that injection of acepromazine alone or in combination with tramadol did not cause any significant changes in the intraocular pressure. Injecting Acepromazine alone to dogs in the first 30 min caused a significant increase and then a decrease in the intraocular pressure, but no significant change occurred in intraocular pressure in the Acepromazine‐tramadol group during this period, probably due to the predominant effect of Acepromazine on tramadol. The same issue was seen in the present study causing a small change in intraocular pressure in the MA group during the first 15 min. In fact, the ascending effect of Acepromazine was dominant compared to the descending effect of Medetomidine at this dose. Tamura et al. (2002) and Stephan et al. (2003) achieved the same results so that the intramuscular administration of Acepromazine‐hydromorphone at 0 to 10 and 25 min after injection showed a reduction in the intraocular pressure, but the reduction was not significant. Results of the present study were consistent with the research by Mrazova et al. (2018), which intravenously injected a similar dose of Medetomidine (10 µg/kg). In their study, the intraocular pressure was measured at 5 and 10 min after injection, indicating a decreasing trend.

The declining trend, similar to this study, was not significant at the 5th min. In a study by Verbruggen et al. (2000), no significant decrease in intraocular pressure was reported after intravenous injection of 45 µg/kg Medetomidine into 14 dogs. Similarly, in the study of Kanda et al. (2015), a significant reduction was only seen at the dosage of 80 µg/kg of Medetomidine. They concluded that changes in the intraocular pressure did not occur at low and medium doses of Medetomidine and only occurred at high doses of the drug. Whereas, a significant reduction in intraocular pressure was seen at 15 and 20 min after injection in this study. So, these results contradicted the conclusion of Kanda et al. (2015) and Verbruggen et al. (2000). Artigas et al. (2012) mentioned similar reasons for the reduction in intraocular pressure after the administration of alpha‐2 agonists. Micieli et al. (2018) examined the separate eye effects of intravenous injection of two drugs, Dexmedetomidine and Acepromazine, on dogs at 2 µg/kg and 0.015 µg/kg during 25 min. In their study, the intramuscular injection of Dexmedetomidine compared with Acepromazine significantly reduced intraocular pressure at 10 and 15 hr after injection. In this study, the reduction was also seen after the muscular administration of Dexmedetomidine. However, the reduction was significant 20 min after administration compared to the baseline time. In addition, alpha‐2 agonists such as Dexmedetomidine and Medetomidine can induce vomiting and subsequently increase the intraocular pressure (Sinclair, 2003). However, it was inconsistent with intraocular pressure measured in an animal case from the DA group that had vomiting during 15–20 min in this study.

In this study, a decrease was seen in pupil diameters in all four groups, but the reduction was only significant in DA and MA groups. In a study by Micieli et al. (2018), which separately examined the visual effects of intravenous injection of 0.015 mg/kg of Acepromazine and 0.002 mg/kg of Dexmedetomidine, there was a significant reduction in pupil diameters in both groups. Similar results were obtained in a study by Artigas et al. (2012) in 2012, after the intravenous injection of 0.005 mg/kg of Dexmedetomidine, and a study by Mrazova et al. (2018), after the separate intravenous injection of 0.01 mg/kg of Medetomidine and 0.02 mg/kg of Acepromazine. According to previous studies, changes in the size of pupil diameter after injection of Dexmedetomidine, Acepromazine and Medetomidine had a decreasing trend, but changes were significant in DA and MA groups in this study, probably due to the synergistic effects of two drugs together on the pupil diameter. Kanda et al. (2015) stated that contrary to previous findings reported by Verbruggen et al. (2000), neither Medetomidine nor Xylazine caused a clear contraction in the pupils; and the inhibition of sympathetic activity by alpha‐2‐adrenoceptor agonists such as Medetomidine and Xylazine inhibited the contraction of mydriasis muscles that were initially innervated by sympathetic nerves. On the other hand, based on studies by Santos et al. (2013) and Kovalcuka and Birgele (2009) that intramuscularly injected 0.1 mg/kg of Acepromazine, the drug significantly decreased the pupil diameter. Therefore, it can be inferred that the reduction in pupil diameter in both DA and MA groups was due to the miotic effect of Acepromazine. Despite the lack of study on the combined effects of the drugs on the pupil diameter, according to results of this study, adding Acepromazine to Dexmedetomidine and Medetomidine could increase the miotic properties of both alpha‐2 agonists because the decreasing trend in D and M groups was not significant in this study, whereas it was significant in DA and MA groups and was probably due to the synergistic effect of Acepromazine with both drugs to reduce the pupil diameter; however, there is a need for further studies to prove the claim.

Based on Schirmer's test, the normal rates of tear secretion in a dog and cat were usually between 15 and 25 millimetres per minute (Birchard & Sherding, 2005). In this study, the tear secretion rate was normal in the D group, but was from 13.87 to 14.65 in the other three groups, and was classified as the slightly reduced group based on the Dodam's scheme. The slight decrease at T0 might be caused by animal examination and handling stress according to Birchard and Sherding (2005). In this study, there was a greater insignificant reduction in tear secretion in the DA group than the D group alone at all three times. A similar result was obtained in the comparison of the MA group with the M group in this study. Based on the results, a further reduction was achieved in the tear secretion when two sedatives such as Medetomidine and Acepromazine or Dexmedetomidine and Acepromazine were administered together. The result was consistent with a study by Sanchez et al. (2006). They found that the combination of Medetomidine‐Butorphanol reduced the tear secretion more than Medetomidine alone. Santos et al. (2013) conducted a similar study and found that prescribing a combination of Acepromazine and tramadol caused a reduction in the tear secretion more than each one alone. Actually, the reduction of tear production after administration of sedatives could be due to their central effects on the neural regulation of tear secretion. Therefore, it was suggested that the rate of tear secretion would be lower in the combination of sedatives and opioids; and the reduction was mostly due to one or more of the following mechanisms: Central effects of drugs on the autonomic regulation of tear secretion, Effective antinociception, Vascular contraction in the lacrimal gland and Changes in metabolism of tear gland cells (Di Pietro et al., 2016; Dodam et al., 1998; Sanchez et al., 2006). In addition, the administration of the sedatives disrupts blinking and increases the evaporation of tears and eventually dry eyes (Leonardi et al., 2019). In our study, the tear secretion decreased in all four groups after the administration of sedatives, and the reduction was significant in the M and MA groups at all three times, +5, +15 and +20 min after intramuscular injection of drugs. Therefore, it can be argued that the combination of two sedative drugs together has a similar effect on further reduction of tear secretion than each one alone.

## 5 CONCLUSION

Since the systemic administration of two compounds, Medetomidine‐Acepromazine and Dexmedetomidine‐Acepromazine decreases the intraocular pressure, their use in animals with corneal injury, corneal perforation, glaucoma and corneal ulcers is not prohibited. Furthermore, since both compounds reduced the amount of tear secretion, especially Medetomidine‐Acepromazine compound, which significantly reduced the amount of tear, it is essential to prescribe tear substitutes to prevent the complications of lower tear secretion after creating the sedation by these drugs.

## CONFLICT OF INTEREST

There is not any conflict of interest.

## AUTHOR CONTRIBUTION

**Ali Aghababaei:** Conceptualization; Data curation; Investigation; Writing‐review & editing. **Ali Baniadam:** Conceptualization; Data curation; Writing‐review & editing.

### PEER REVIEW

The peer review history for this article is available at https://publons.com/publon/10.1002/vms3.467.
